# Integrating Sex and Gender into an Interprofessional Curriculum: Workshop Proceedings from the 2018 Sex and Gender Health Education Summit

**DOI:** 10.1089/jwh.2018.7339

**Published:** 2019-12-10

**Authors:** Basmah Safdar, Angela F. Jarman, Rebecca Barron, Daniel H. Gouger, Tess Wiskel, Alyson J. McGregor

**Affiliations:** ^1^ Department of Emergency Medicine, Yale School of Medicine, New Haven, Connecticut.; ^2^ Department of Emergency Medicine, University of California-Davis, Sacramento, California.; ^3^ Department of Emergency Medicine, Portsmouth Regional Hospital, Portsmouth, New Hampshire.; ^4^ Department of Anesthesiology, Virginia Commonwealth University Health System, Richmond, Virginia.; ^5^ Team Health Northeast Group, Emergency Physician Special Operations, Waterville, Maine.; ^6^ Department of Emergency Medicine, Alpert Medical School of Brown University, Providence, Rhode Island.

**Keywords:** sex, gender, curriculum, SMART, milestones

## Abstract

***Background:*** In the last 3 years, the National Institutes of Health (NIH) declared advancement of understanding the role sex as a biological variable has in research a priority. The burden now falls on educators and clinicians to translate into clinical practice the ensuing body of evidence for sex as a biological variable that clearly shows the effect of sex/gender on disease diagnosis and management. The 2018 Sex and Gender Health Education Summit (SGHE) organized an interdisciplinary and interprofessional workshop to (1) analyze common clinical scenarios highlighting the nuances of sex- and gender-based medicine (SGBM) in presentation, diagnosis, or management of illness; (2) utilize valid educational and assessment tools for a multiprofessional audience; and (3) brainstorm standardized learning objectives that integrate both.

***Materials and Methods:*** We describe the iterative process used to create these scenarios, as well as an interprofessional forum to develop standardized SGBM case-based objectives.

***Results:*** A total of 170 health education professionals representing 137 schools of Medicine, Dentistry, Pharmacy, Public Health, Nursing, Physical, and Occupational Therapy participated in this workshop. After attending the workshop, participants reported a significant increase in comfort level with using diverse educational modalities in the instruction of health profession learners. Recurrent themes included case-based learning, use of sex-neutral cases, simulation, and standardized patient scenarios for educational modalities; and self-assessment, peer assessment, and review of clinical documentation as used assessment tools. Materials created for the workshop included teaching SGBM case scenarios, methods of assessment, and sample standardized objectives.

***Conclusion:*** The SGHE Summit provided an interdisciplinary forum to create educational tools and materials for SABV instruction that may be applied to a diverse audience.

## Introduction

Over the past two decades, a rapidly growing body of literature has established the influence of sex and gender on the presentation, diagnosis, management, and prognosis of disease processes, and therefore must be incorporated into routine clinical care.^1^ This movement gained momentum in 2016 when the National Institutes of Health (NIH) mandated the inclusion of biological sex in every federally funded basic science research study.^2^ The resulting attention to inclusion of sex and gender in medical research has been encouraging.^3^ However, these advancements will not change patients' lives unless they are translated into clinical knowledge for the providers directly delivering their care. The 2018 Sex and Gender Health Education Summit (SGHE) convened a large multiprofessional group of stakeholders, including educators, students, researchers, policy makers, administrators, and representatives of funding agencies to help bridge this gap.

Translation of the science of sex and gender research aligns well with an increasing interest in precision medicine, which explores how treatment or prevention approaches for any disease can be modified for a given cohort (such as males or females) or even large-scale populations, based on the combination of genetic, molecular, environmental, and social factors that are unique to those populations.^4^ However, such translation is often hindered by lack of access to subject matter expertise and inadequate understanding of effective and innovative educational tools by which to deliver this content.^5^ Health professions increasingly are adopting educational models tied to entrustable professional activities and competencies.^6,7^ To achieve this task, curriculum developers and reformers need effective mapping processes that support data collection and the creation of quality benchmarks.^8^ However, the absence of a standardized vocabulary across health professions' education has been a considerable challenge in this effort.^9^ The field of research in medical health sciences education has rapidly grown in the past several years to now include standardized definitions, delivery methods, and assessment tools from the Association of American Medical Colleges, but they seem to remain an underutilized resource.^10^ There is also wide variation between professions in the methods of dissemination of sex- and gender-specific research findings relevant to that profession. A multistakeholder forum was therefore needed to utilize the diverse expertise of the group in brainstorming the sex- and gender-based medicine (SGBM) nuances relevant to clinical care, using optimally measurable and standardized delivery methods.

For the 2018 SGHE, we organized an interdisciplinary and interprofessional workshop to achieve the following goals: (1) to analyze four common clinical scenarios to highlight the sex and gender nuances in presentation, diagnosis, or management of illness; (2) to utilize standard educational and assessment tools to deliver these SGBM medicine clinical pearls to a multiprofessional audience; and (3) to brainstorm two to three learning objectives using these discussions for each case that integrates multiprofessional clinical and educational tools. We also aimed to use the forum to describe challenges unique to different professions as well as share creative solutions to integrate sex and gender into their instructional methods within the classroom.

## Materials and Methods

The SGHE Summit Workshop Planning Committee used an iterative process to identify four SGBM clinical scenarios relevant to an interprofessional audience preworkshop. These scenarios informed the creation of gender-neutral patient encounters and utilized evidence-based sex/gender guide points for the presentation, diagnosis, and management of the illness. The same themes were used to facilitate the discussion for creating inclusive interprofessional sex- and gender-based educational learning objectives and assessment methods for each scenario during the workshop.

The development of the workshop included a preworkshop production phase and facilitator training.

### Preworkshop production phase

#### Multiprofessional clinical case development

An iterative process was used to develop four clinical scenarios that highlighted SGBM evidence most relevant for a multiprofessional discussion at the workshop. Two committee members (A.F.J. and B.S.) generated the first list of clinical topics (*n* = 44). This list was then vetted by the Workshop Planning Committee and the SGHE Summit Executive Planning Committee that was representative and inclusive of the professions participating in the Summit (reference main summit proceedings). Four clinical topics were selected as having broad relevance for Summit participants. These topics were then further developed into gender-neutral cases that highlight sex and gender differences in the presentation, diagnosis, and management of the condition along with discussion points and references. The full cases were made available for review to participants on the Summit smart phone application before the Summit (Supplementary Appendix SA1). Shorter print versions were disseminated to participants during the workshop.

#### Standardized educational and assessment tools, an interprofessional lens

We provided all participants the MedBiquitous Standardized Curriculum Inventory as reference for educational tools and assessment methods.^10^ A 2016 initiative by the AAMC MedBiquitous Curriculum Inventory Working Group, this tool enables standardization by providing succinct, universal curricular definitions that easily extend into the learning environment for course instructors and developers.^10^ The workshop participants selected salient clinical points from one of the four cases that were provided and matched them to a logical instructional and assessment method in the inventory that best matched the needs of the learners in their professions. Participants were challenged to select instructional and assessment methods that they use less frequently to promote innovation and expand their educational armamentarium. Workshop facilitators prompted interprofessional discussion about unique opportunities and challenges that exist in specific learning environments and with particular learners across the health professions continuum, which might warrant selection of certain methods over others to facilitate learning in cognitive, psychomotor, and/or affective domains.

### Facilitator training

Before the workshop, the Summit co-chairs Dr. Marjorie Jenkins and Dr. Alyson J. McGregor conducted three facilitator training sessions for all facilitators, using standardized facilitator and participant guides (Supplementary Appendix SA2).

### Multistakeholder workshop

To facilitate a collaborative discussion, the workshop organizers chose discussion groups of 8–12 participants with at least one representative from each of participating health professions.

Workshop facilitators at each table prompted the group to select one to two cases and to abstract the SGBM clinical nuances of that case, with the goal of creating succinct SMART objectives, as described below.

### SMART objectives

SMART learning objectives, which grew from management world concepts aimed at improving organizational strategy,^11^ make teaching practical by giving it a sense of focus and direction. SMART objectives have also been shown to increase knowledge acquisition for learners and promote behavior and performance change.^12,13^ SMART objectives are as follows:

- Specific: Objectives should identify who will do how much of what, how well.
- Measurable: Instruction should be designed with specific assessment methods in mind.
- Attainable: Anticipated levels of change in learners should be consistent with both resource and time constraints.
- Relevant: The instruction, assessment, and change in learners should be relevant to their stages of training and to the subject matter.
- Time bound: Objectives should articulate a clear and reasonable time frame within which to be accomplished.

Workshop facilitators were provided with a SMART Learning Objective Guide (Supplementary Appendix SA3). Using salient clinical points from the four SGBM cases and by selecting instruction and assessment methods from the MedBiquitous Standardized Curriculum Inventory, participants were asked to write SMART learning objectives as an exercise in the incorporation of SGBM into existing course curricula. Facilitators challenged workshop participants to modify SMART learning objectives based on how sex and gender variables could change intended learning outcomes. Facilitators also asked the group to share barriers and solutions to integrating sex and gender into their instructional methods within the classroom, including considering institutional resources, faculty attitudes, and availability, and how sex and gender might fit into the overarching curriculum.

### Framework of discussions

**FIG. 1.**
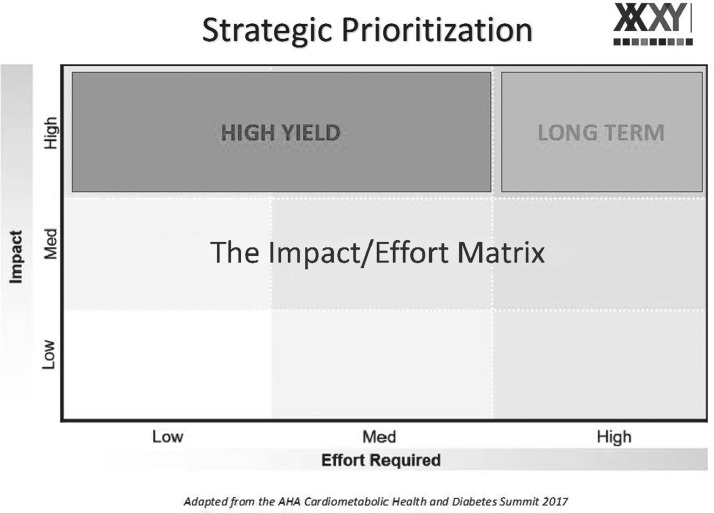
Framework for strategic prioritization of workshop discussions.

Facilitators encouraged participants to brainstorm optimal solutions that strategically prioritized two types of solutions (Fig. 1):

1. High-impact, low-effort solutions: these were high-impact creative instructional methods that would require fewer resources, as well as less effort to plan or organize at an individual level or departmental level (*e.g.*, adding an objective to an already developed didactic that incorporates the influence of sex and gender on the topic).
2. High-impact, high-effort solutions: these were high-impact solutions that required more resources, as well as more effort to organize and plan, but aimed to change systems at an institutional or national level for the most effective long-term solutions.

For example: to integrate sex and gender throughout medical student curricula or incorporating relevant questions in standardized national testing.

### Workshop assessment

Before the commencement of the workshop, participants were asked to complete a three-question preworkshop survey to assess ease with integrating sex and gender topics into educational curricula (Table 1). At the completion of the session, participants answered the same three questions. Descriptive analyses were used to calculate percentage responses to preworkshop and postworkshop survey questions.

**Table 1.** Preworkshop and Postworkshop Assessments by Question

*Survey item*	*Assessment question*	*Preworkshop (%)*	*Postworkshop (%)*
1	I am able to apply SGBM knowledge to common clinical scenarios in terms of presentation, diagnosis, and management **(**Strongly agree**)**	13.7	28.9
2	I am able to utilize diverse educational modalities in the instruction of health professions learners on SGBM **(**Strongly agree**)**	15.1	34.8
3	I am able to prioritize highest yield instructional strategies to incorporate SGBM into the instruction of health professions learners **(**Strongly agree**)**	6.2	20.7

SGBM, sex- and gender-based medicine.

Following the completion of the workshop, two authors (A.F.J. and T.W.) collated facilitator and scribe notes from each small group and organized them by case and domain (clinical pearls, educational methods, assessment methods, and SMART objectives). We applied codes for iterative ideas and quantified the number of times each code was used. Coding was performed by the same two authors together to ensure agreement in the application of codes. We used a grounded theory approach to induce common themes from the data both within and between cases.^14^ In addition, noncategorical innovative ideas were separately recorded.

## Results

A total of 170 health education professionals participated in the SGHE Summit. Among those participants were representatives from schools of Medicine, Dentistry, Pharmacy, Public Health, Nursing, Physical, and Occupational Therapy. The participants represented 137 academic institutions across the United States as well as Canada, South Korea, and Ghana. Of the participants, 145 of 170 completed the survey, giving a response rate of 85%. We assessed preworkshop and postworkshop comfort with applying SGBM knowledge, which is summarized in Table 1.

Each of the four case scenarios facilitated a robust discussion on the clinical nuances that resonated with different professions. Each table chose one or both cases available to them. Both within and between cases, there were a number of recurrent themes, the most common of which are summarized in Tables 2–5. Sample SMART objectives that were generated for each case are also included in Tables 2–5.

**Table 2.** Leading Clinical and Educational Themes for Metabolic Case

Supplementary Appendix SA1 (Case 1)	27-Year-old previously healthy patient with new-onset diabetes mellitus
Sex and gender clinical pearls	1. Social determinants of health (disparities, stress, diet, culture, exercise, psychosocial factors, women prepare food, and “fat shaming”)
	2. Differential risk attribution (of traditional vascular risk factors, *e.g.*, diabetes, smoking, and nontraditional risk factors such as mental health and chronic stress)
	3. Individualization of treatment strategies (individualized preventive strategies, different success of weight loss plans, and gender-nuanced motivational interviewing)
	4. Differences in management and pharmacotherapy
Standardized education methods	1. Case-based learning
	2. Observed Standardized Clinical Encounter (OSCE)
	3. Standardized patient
	4. Simulation
Standardized assessment methods	1. Self-assessment
	2. Clinical documentation or care plan
	3. Peer assessment
	4. Test or quiz
Sample SMART objective	At the end of this course, students will be able to identify two evidence-based differences in the treatment of type 2 diabetes based on biological sex, as measured by precourse and postcourse surveys.

**Table 3.** Leading Clinical and Educational Themes for Neurologic Case

Supplementary Appendix SA1 (Case 2)	74-Year old with evolving left middle cerebral artery stroke
Sex and gender clinical pearls	1. Differential risk attribution (vascular risk factors)
	2. Differences in treatment (tpa and asa)
	3. Institutional bias
Standardized education methods	1. Simulation
	2. Video/online asynchronous
	3. Standardized patients
	4. Case-based learning
Standardized assessment methods	1. Online quiz
	2. Peer assessment
	3. Short answer questions
	4. Developing an SGBM-based treatment plan
Sample SMART objective	During the simulation, 90% of students will ask sex- and gender-specific questions when obtaining medical history, as measured by assessment of their peers.

**Table 4.** Leading Clinical and Educational Themes for Dental/Pain Case

Supplementary Appendix SAP1 (Case 3)	37-Year old with dental fracture and acute pain
Sex and gender clinical pearls	1. Implicit bias (provider perceptions/anchoring bias, discrimination)
	2. Social determinants of health (compliance, economics, neighborhoods and safety, access to care, and use of services)
	3. Pharmacology differences (pharmacokinetics/dynamics)
	4. Tailoring treatment based on sex and gender (frequent undertreatment in women and link to chronic pain)
Standardized education methods	1. Case-based learning
	2. Observed standardized clinical exams (OSCEs)
	3. Add sex and gender to existing educational methods
	4. Literature review
Standardized assessment methods	1. Self-assessment/reflection
	2. Peer assessment
	3. Direct observation and assessment
	4. Creating care plans
Sample SMART objective	After completing clinical cases, learners will demonstrate clinical proficiency in sex- and gender-based implications of administering opioids for acute and chronic pain through creating care plans demonstrating dosing, side effects, and risk for addiction and chronic pain.

**Table 5.** Leading Clinical and Educational Themes for Cardiovascular Case

Supplementary Appendix SA1 (Case 4)	58-Year old with shortness of breath acute coronary syndrome
Sex and gender clinical pearls	1. Differential risk attribution (traditional and nontraditional coronary risk factors)
	2. Social determinants of health (socioeconomic status, access to care, time, money, and resources)
	3. Sex and gender differences in treatment plans (less aggressive in women, medication treatments differ, and behavior change)
	4. Understand presentation differences (delay in presentation and various symptoms)
Standardized education methods	1. Simulation
	2. Case-based learning
	3. Journal club
	4. Standardized patients
Standardized assessment methods	1. Peer assessment
	2. Assessment by rubric
	3. Direct observation and evaluation
	4. Self-assessment
Sample SMART objective	At the end of the patient simulation experience, learners should be able to describe three sex-specific differences in the presentation of acute myocardial infarction as evaluated by postexperience quiz

### Summary of themes from facilitated discussions

Across all cases and groups, the need for an interdisciplinary approach was emphasized. Participants were clear that to provide individualized and sex- and gender-focused care, an interdisciplinary lens is imperative. Recurrent themes for SGBM content, educational tools, and assessment methods are summarized in Tables 2–5.

#### SGBM themes

Three out of four cases found social determinants of health to be of critical importance. These were typically influenced by patient gender and included economic concerns, access to health care, caregiver stress, diet, cultural expectations, access to exercise, and psychosocial factors. The prevalence of risk factors predictive of diseases and their sex-based differential risk attribution in women and men was also emphasized as important clinical pearls, particularly in relationship to vascular risk. In addition, each group emphasized the sex differences in diagnostics (sensitivity and specificity) and pharmacologic management (in effectiveness and adverse events), and gender differences in motivational counseling for weight loss or exercise as important clinical pearls.

#### Themes for educational methods

Thematic trends in preferred educational methods for teaching SGBM content were also evident. Case-based learning was popular across all groups, and many groups noted the importance of using sex-neutral or sex-interchangeable cases in teaching. This would mean using the same clinical scenario and separately discussing with a female and male patient to highlight SGBM differences in presentation, diagnosis, and treatment of the disease. Simulation and standardized patient scenarios were also repeatedly suggested as educational modalities that paired with good assessment tools. Participants identified use of journal clubs/literature review to be “low-resource, high-impact” method for learners to incorporate SGBM in their curricula. Each of these modalities is designed to put the learner “on the spot” and provide experience with asking and talking about sex and gender with patients.

#### Themes for assessment

Self-assessment was commonly suggested as a standardized assessment method. This included a range of methods including, narrative assessment, self-evaluation, and self-reflection. Participants felt that this would be a valuable tool to identify and reflect on affective domains, including implicit biases related to sex and gender. Participants also frequently suggested peer assessment in observed clinical encounters as a useful tool both for the learner and the peer. Finally, treatment plans and related clinical documents were also preferred standardized assessment methods and the need to address interdisciplinary concerns was emphasized.

#### Novel themes

Most of the recurrent themes were teaching and assessment methods that have been tried and tested. However, the strength of an interprofessional forum was that it also yielded insights into novel methods tested by individual sites or professions. Examples included the following: creation of an asynchronous online module offering SGBM content (*e.g.*, video module)^15^; longitudinal case-based learning; five-stage simulation with different professions based on sex/gender scenario; online games that incorporate SGBM content; online quiz bank for SGBM questions; and intersectionality of race, culture, and gender influences on clinical care in addition to sex-based differences. Novel assessment methods included postcourse narrative assessment with reflection; rubric assessment for standardization; survival in games; and use of implicit bias tool. A “high-yield, low-resource” step was to include sex/gender as an objective to already prepared lecture or presentation and to reflect the salient features of the lecture through this lens.

#### Barriers and solutions for integration

This workshop also yielded insights on possible barriers to implementation of SGBM-focused curricula, including the need for peer education and faculty development. Participants noted that they would likely need to “educate the educator” in many instances, where SGBM knowledge is not widely shared among their peers. Investing the time to create new educational materials and assessment methods was also considered added deterrents. Possible solutions included identifying one to two SGBM champions at each institution, who would form core teaching faculty as well as create a repository of teaching cases with SGBM-related clinical pearls and standardized assessment methods.^16^ Participants emphasized the importance of gaining institutional support and noted that positioning one's institution as a leader in higher education and as an early adopter of this new knowledge is helpful in engaging leadership stakeholders.

## Conclusion

The 2018 SGHE Summit brought together multiple stakeholders who were engaged in a workshop whose purpose was to demonstrate the integration of sex and gender into an interprofessional curriculum, with workshop participants successfully accomplishing the following goals: (1) synthesize the breadth of evidence that adds to the understanding of sex- and gender-specific health in a variety of clinical scenarios; (2) utilize current active learning educational modalities to demonstrate inclusion of sex and gender into existing curricula; (3) create SMART objectives that demonstrate learning and assessment of sex- and gender-inclusive content; and (4) create a framework for initiating an integrative curricular change that is pertinent to specific professions.

The methods described here serve as a template for institutional groups of curricular leaders, faculty, and student groups to begin the process of integration of sex and gender into educational curricular objectives and assessments that are relevant to their practice and profession. The workshop highlighted the importance of using a multiprofessional forum in integrating the science of sex and gender into mainstream curricula as a step toward personalized patient care. One of the challenges identified in such a forum was the variability in advances in educational methods in respective professions. As a result, the recurrent themes primarily included more tried and tested methods. However, in this article, we also identified some of the novel methods that were brought up during the discussions to aid an interprofessional audience. We describe a step-by-step process toward successfully addressing the challenges toward achieving these goals. Finally, the workshop helped create educational materials, including clinical scenarios with evidence-based SGBM pearls as well as SMART objectives that are available for immediate use to the readers.

## Supplementary Material

Supplemental data

Supplemental data

Supplemental data

## Supplementary Material

Supplementary Appendix SA1

Supplementary Appendix SA2

Supplementary Appendix SA3
