# Supplementary material for: Integrating Sex and Gender into an Interprofessional Curriculum: Workshop Proceedings from the 2018 Sex and Gender Health Education Summit
Source: J Womens Health (Larchmt). 2019 Dec 10;28(12):1737–42. doi: 10.1089/jwh.2018.7339 (PMC6919237; doi:10.1089/jwh.2018.7339)
Supplement: Supplemental data [file Supp_Appendix1.docx]

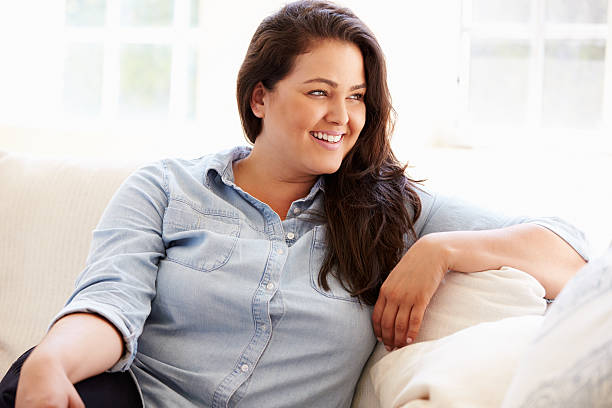
**
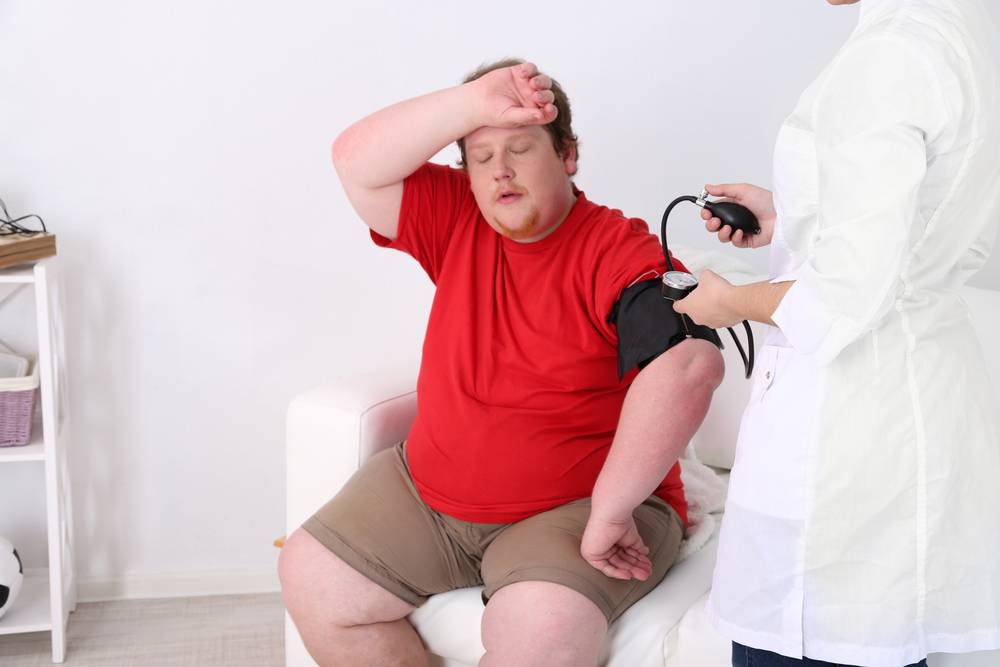
**

**Case 1: Metabolic**

27 year old presenting with increased urination

PMH: none, no meds, no allergies

FH: diabetes type 2, congestive heart failure, hypertension, coronary artery disease

SH: Smoker (1/2ppd), occasional marijuana

VS: 107 BP 143/79 T 36.6 RR 15 O2 92%, BMI: 42

**Presentation:** This patient presents to the clinic complaining of increased urination for several days. On review of systems, also endorses increased thirst and lightheadedness. The patient states that they do not have any prior medical history and does not have a primary care provider. On exam, the patient has central obesity and you note acanthosis and numerous skin tags on the trunk.

**Diagnosis:** new onset diabetes mellitus, type II (T2DM)

**Discussion Points**

1. Epidemiology- metabolic syndrome affects 20-30% of population
   1. In <50 years old, higher rates of metabolic syndrome in men, while after 50 years it is higher in women
   2. No clear sex differences in DM prevalence
2. Presentation & Diagnosis
   1. T2DM diagnosed at lower age and BMI in men than women
   2. Women more likely to be diagnosed by oral glucose tolerance test vs fasting glucose measurements
3. Risk factors
   1. Psychosocial risks greater in women for T2DM as well as stigmatization
   2. Women with higher rates of obesity and greater impact of reproductive factors on diabetes
   3. Family history of diabetes confers higher risk for women than men
   4. T2DM has greater increases in CV, MI and CVA risk in women
4. Management
   1. Lifestyle changes (exercise & weight loss): men may be better responders
   2. Women with lower success of glucose lowering therapy and dual therapy and higher rates of hypoglycemia on insulin
   3. Women with impaired fasting glucose may respond to lifestyle and pharmacologic management, with attention to psychosocial problems
   4. DM is an important risk factor for cardiovascular disease, though the association is much stronger for women; risk of fatal CAD associated with DM2 is 50% higher in women
   5. Women with DM receive less aggressive treatment compared to men, e.g. less likely to be prescribed ASA, beta blockers, statins
5. Pregnancy Considerations
   1. Increasing rates of gestational diabetes (GDM) in same populations as T2DM: obesity, increasing age, ethnic minorities
   2. GDM leads to higher rates of pre-eclampsia, cesarean as well as DM later in life- estimates of 15- 70% of women with GDM later develop DM
   3. GDM also an independent risk factor for CV disease later in life
   4. Offspring of women with GDM also more likely to develop complications including macrosomia, neonatal hypoglycemia, hyperbilirubinemia and birth trauma
   5. Intrauterine hyperglycemia linked to maternal transmission of DM

**References:**

ACOG Practice Bulletin No. 190: Gestational Diabetes Mellitus. Committee on Practice Bulletins—Obstetrics. Obstet Gynecol. 2018 Feb;131(2):e49-e64. doi: 10.1097/AOG.0000000000002501.

Barrett-Connor et al. Why is diabetes mellitus a stronger risk factor for fatal ischemic heart disease in women than in men? The Rancho Bernardo Study. JAMA. 1991 Feb 6;265(5):627-31.

DECODE Study Group: Age- and sex-specific prevalence of diabetes and impaired glucose regulation in 13 European cohorts. Diabetes Care 2003; 26: 61-69

Ding EL et al. Sex differences of endogenous sex hormones and risk of type 2 diabetes: a systematic review and meta-analysis. JAMA 2006; 295; 1288-1299.

Huxley, R et al Excess risk of fatal coronary heart disease associated with diabetes in men and women: meta-analysis of 37 prospective cohort studies. BMJ 2006: 332:73-78

Huxley et al. Cigarette smoking as a risk factor for coronary heart disease in women compared with men: a systematic review and meta-analysis of prospective cohort studies. Lancet. 2011 Oct 8;378(9799):1297-305

Kautzky-Willer et al. Sex and gender differences in therapy of type 2 diabetes. Diabetes Res Clin Pract. 2017 Sep;131:230-241.

Kautzky-Willer et al. Sex and Gender Differences in Risk, Pathophysiology and Complications of Type 2 Diabetes Mellitus. Endocr Rev. 2016 Jun;37(3):278-316.

Metzger BE, et al: International association of diabetes and pregnancy study groups recommendations on the diagnosis and classification of hyperglycemia in pregnancy. Diabetes Care 2010; 33; 676-682.

Mogelvang R, Scharling H, Jensen JS. A simple linear model for the effect of changes in metabolic risk factors on incident of coronary heart disease. J Int Med 2006; 259:561-8.

Pucci et al. Sex- and gender-related prevalence, cardiovascular risk and therapeutic approach in metabolic syndrome: A review of the literature. Pharmacological Research 2017: 120:34-42.

Yusuf S et al: Effect of potentially modifiable risk factors associated with myocardial infarction in 52 countries (the INTERHEART study); case-control study. Lancet 2004; 364; 937-952


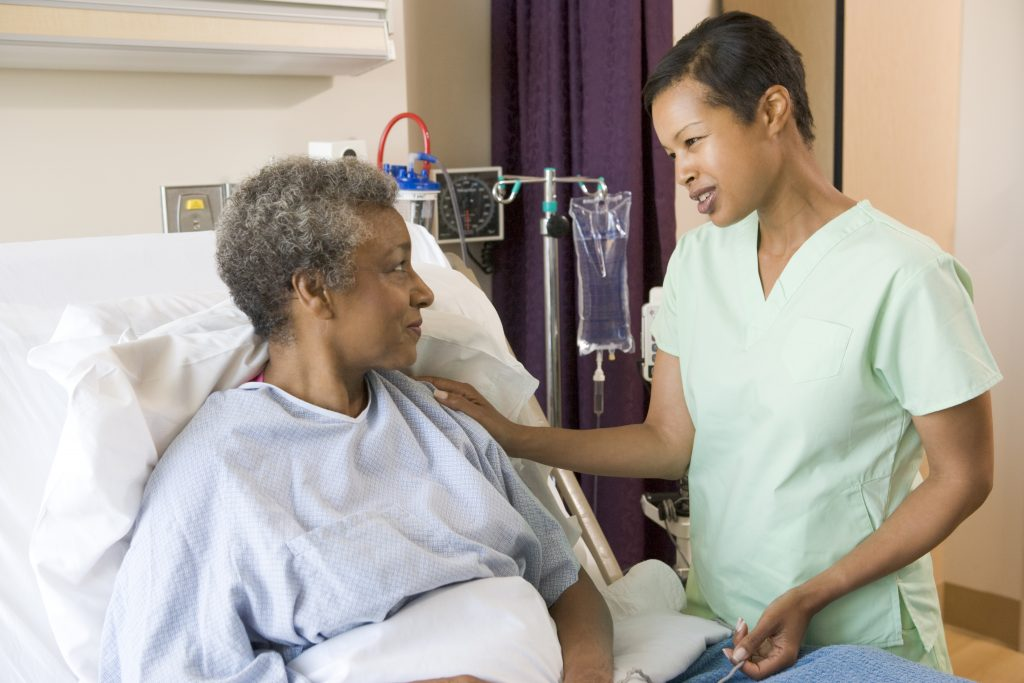
 **
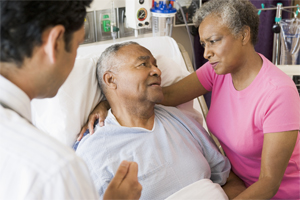
**

**Case 2: Neurologic**

74 year old with sudden word finding difficulty

PMH: HTN, HLD, PVD on clopidrogel and aspirin

SH: prior smoker (40 pack year)

FH: Mom with stroke in 50s, brother with cardiac stents

VS: HR 89 BP 178/73 T 36.9 RR 22 O2 94%

**Presentation:** This patient was with a family member at lunch when they began having difficulty speaking and finding words. Since that time the patient has been unintelligible to family. The patient’s last known normal was 2.5 hours prior to arrival. On exam, you find R arm weakness in addition to marked dysarthria and fluctuating expressive aphasia.

**Diagnosis**: Evolving left middle cerebral artery CVA

1. Epidemiology: Women have higher lifetime risk of stroke but tend to have them later in life
2. Presentation: Women tend to have more “atypical” symptoms
   1. May present as pain, change in level of consciousness, and non neurologic symptoms
   2. Women also tend to present later and tend to live alone given longer life expectancy
3. Risk Factors:
   1. Women more likely to have hypertension, atrial fibrillation (also less likely to be anticoagulated for it); diabetes and metabolic syndrome increase stroke risk disproportionately in women
   2. Men more likely to have atherosclerotic disease, diabetes, CAD
   3. ASA effective in primary prevention in women, not in men
4. Diagnosis
   1. Women are less likely to receive timely CT scan and physician evaluation. Women less likely to receive echocardiography and carotid ultrasonography during their workup
5. Pregnancy Considerations:
   1. Preeclampsia, eclampsia, htn of pregnancy, and gestational dm all increase stroke risk for years after pregnancy
6. Pathophysiology: Estrogen as a neuroprotectant
   1. In animal models, estrogen down regulates inflammation, reduces cell death, causes vasodilation in cerebral vessels
7. **Management**:
   1. Women up to 30% less likely to receive IV thrombolytics, even when controlled for delays to presentation and CT scan
   2. Women are found to have a more favorable response to treatment: 10% more likely to be able to perform activities of daily living at 90 days compared to men
8. Prognosis: No differences in case fatality by sex
   1. Women do worse functionally than men after stroke
   2. Women more likely to be disabled and have lower health related quality of life

**References:**

Bushnell CD et al, Advancing the study of stroke in women: Summary and recommendations for future research from an NINDS-Sponsored Multidisciplinary Working Group. *Stroke: Journal of Cerebral Circulation* 2006; 37(9);2387-2399

Di Carlo A et al, Sex Differences in the clinical presentation, resource use, and 3-month outcome of acute stroke in Europe: Data from a multicenter multinational hospital-based registry. *Stroke*: *A Journal of Cerebral Circulation* 2003; 34(5);1114-1119

Gray LJ et al, Sex differences in quality of life in stroke survivors: Data from the Tinzaparin in Acute Ischaemic Stroke Trial (TAIST). *Stroke: Journal of Cerebral Circulation* 2007; 38(11):2960-2964

Kelly AG et al, Predictors of rapid brain imaging in acute stroke: Analysis of the Get with the Guidelines-Stroke Program. *Stroke: Journal of Cerebral Circulation* 2012; 43 (5):1279-1284

Kent DM et al, Sex-based differences in response to recombinant tissue plasminogen activator in acute ischemic stroke- A pooled analysis of randomized clinical trials. *Stroke: Journal of Cerebral Circulation* 2005; 36(1):62-65

Labiche LA, Chan W, Saldin KR et al, Sex and acute stroke presentation. *Annals of Emergency Medicine* 2002; 40 (5):453-460

Reeves MJ et al, Sex Differences in stroke: Epidemiology, clinical presentation, medical care, and outcomes. *The Lancet Neurology* 2008; 7 (10); 915-926

Reeves MJ et al, Quality of care in women with ischemic stroke in the GWTG program. *Stroke: Journal of Cerebral Circulation* 2009; 40(4):1127-1133

Roquer J et al, Sex Differences in first-ever acute stroke. *Stroke: Journal of Cerebral Circulation* 2003; 34(7):1581-1585

Tanaka Y et al, Factors influencing pre-hospital delay after ischemic stroke and transient ischemic attack. *Internal Medicine* 2009; 48(19):1739-1744

**
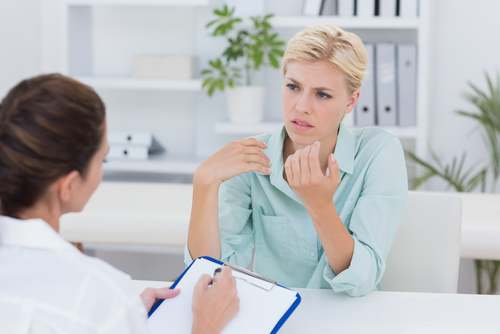

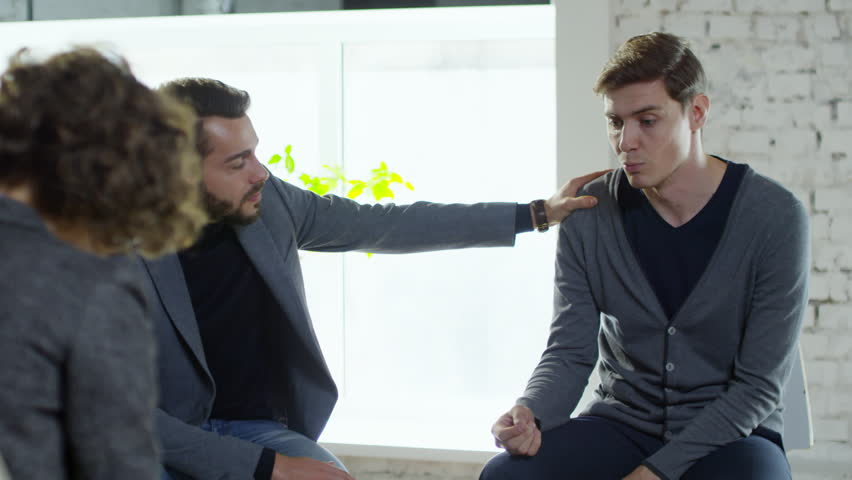
**

**Case 3: Pain/Dental**

37 year old patient with dental pain

PMH: Irritable Bowel Syndrome, Anxiety, Depression

SH: Rare social alcohol, no recreational drugs, non-smoker

FH: Asthma, T2DM

VS: 122 BP 163/94 T 36.8 RR 25 O2 100%

**Presentation:** This patient was eating an apricot yesterday and bit into the pit. The patient was seen by your colleague yesterday and diagnose with an Ellis class II fracture of tooth #31. Yesterday it was sealed and he/she received an inferior alveolar nerve block with good result. Your exam is consistent with this diagnosis and no new complications are evident. The patient has been taking 800mg Ibuprofen and 1g Tylenol with minimal relief. Today is Saturday and there are no dental clinics open until Monday. He/she is tearful and rocking in a recliner in your sick clinic.

**Diagnosis:** Dental Fracture, Acute Pain

**Discussion Points:**

1. Epidemiology: Women suffer proportionally more chronic pain conditions than men (testosterone thought to be protective)
   1. Pain is a complex interplay of biopsychosocial effects (post puberty)
2. Presentation: Acute Pain/Pain Sensitivity
   1. Women generally with greater pain sensitivity and willingness to report pain
   2. Gender and cultural factors also play a role in expression of pain.
3. Risk Factors:
   1. Chronic Pain: Linked with inadequately treated acute pain
   2. Women suffer more painful autoimmune conditions
   3. Women may progress to dependence more quickly
4. Diagnosis
   1. Sex differences in severity of pain difficult to determine due to subjectivity
   2. Women’s pain more likely to be dismissed as psychosomatic
5. **Management**
   1. Pharmacology: Women and men metabolize drugs differently due to hormonal influences, different proportional body fat percentages (larger Vd)
   2. Women tend to have greater anesthesia at same dose of opioid
   3. Women less likely to receive pain meds and wait longer to receive them
   4. Women at greater risk of adverse effects (physiologic & emotional)

**References:**

J. Bartley and R. B. Fillingim. Sex differences in pain: a brief review of clinical and experimental findings. E. British Journal of Anesthesia 111 (1): 52–8 (2013)

Todd, KH et al. Pain in the Emergency Department: Results of the Pain and Emergency Medicine (PEMI) multicenter study. Journal of Pain, 2007. 8 (6): 460-6

Robinson, ME et al. Gender role expectations of pain: Relationship to sex differences in pain. Journal of Pain, 2001. 2 (5): 251-7

Bodnar, RJ and B Kest. Sex differences in opioid analgesia, hyperalgesia, tolerance and withdrawal: Central mechanisms of action and roles of gonadal hormones. Hormones and Behavior, 2010. 58 (1): 72-81.

Robinson, ME et al. Altering gender role expectations: Effects on pain tolerance, pain threshold, and pain ratings. Journal of Pain, 2003. 4(5): 284-8.

**
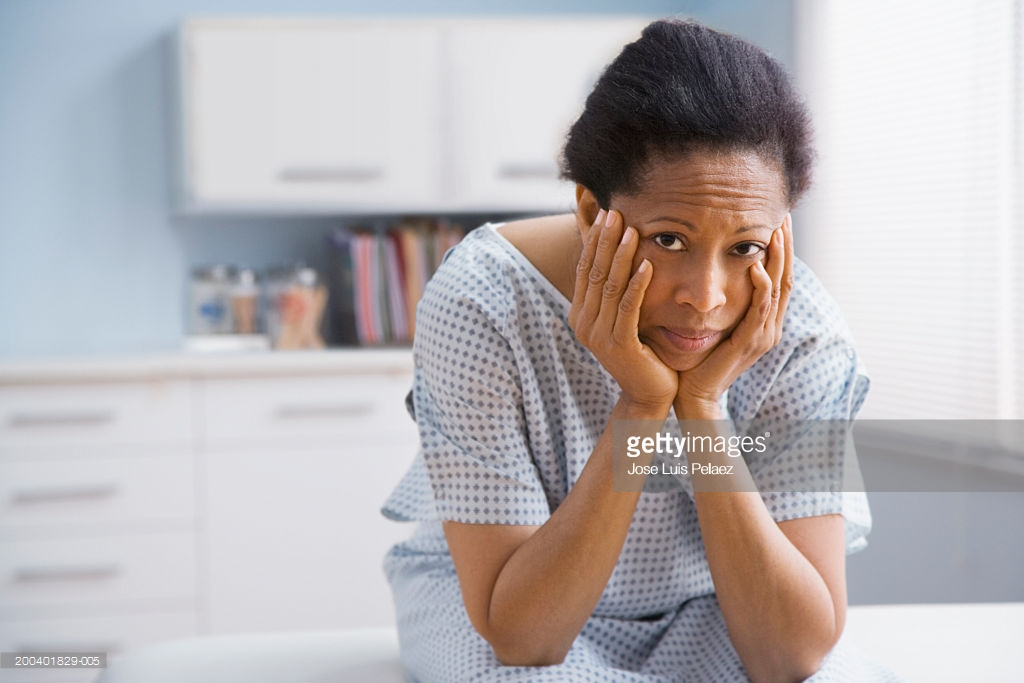

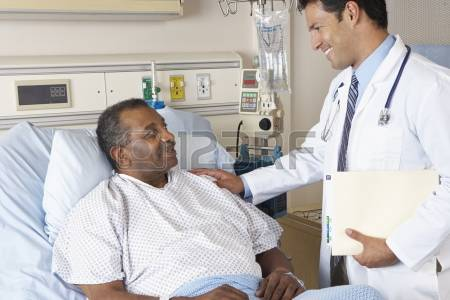
**

**Case 4: Cardiovascular**

58 year old presenting with dyspnea

PMH: HTN on ace-I, HLD on statin, T2DM on metformin (recent A1C 7.2)

SH: Lifelong nonsmoker, social ETOH user

FH: 2 of 2 brothers with coronary stents (<55 years), mom with CHF on “water pill”

VS: HR 67 BP 128/76 T 37.0 RR 17 O2 97%

**Presentation:** This patient presents to an emergency department at the urging of a family member, due to progressive exertional dyspnea. The patient notes this has been ongoing for approximately a week but today developed chest discomfort at rest after a stressful day at work. The patient has never experienced this before but does complain on ROS of generalized fatigue. There are no notable exam findings, and in reviewing the patient’s triage ECG, you note non-specific diffuse T wave flattening.

**Diagnosis:** NSTEMI with mildly elevated troponin, nonobstructive on cath (MINOCA – coronary artery vasospasm, embolization or microvascular dysfunction)

**Discussion Points:**

1. Epidemiology- CAD most common cause of death in women and men in the US
   1. NHANES study showing an increase in mortality in women in midlife from CAD, while declining in men
2. Presentation
   1. Chest pain is the most common presenting complaint for MI in both men and women.
   2. However, women present with more prodromal and atypical symptoms including SOB, weakness and fatigue i.e. with a cluster of symptoms than men.
   3. Women often delay in seeking care for chest symptoms compared to men.
   4. Coronary artery obstruction most common cause of ischemic chest pain in men and women
3. Risk factors
   1. Significance of DM as risk factor- increasing in both men and women
   2. Both DM and smoking increase CV risks in women more than men, and potentially also metabolic syndrome
   3. HTN and HLD increase CAD risk in men more than
   4. Nontraditional risk factors more important in women including depression and autoimmune conditions
   5. Recent decline in vascular risk factors in men (smoking, HTN, HLD) but not in women
4. Diagnosis
   1. ST changes are similar in men and women though women have more T-wave inversions and a potential lower troponin threshold
   2. Risk stratification scores: Reynolds risk score incorporating CRP and metabolic syndrome can be more sex specific than more traditional scores
   3. Cut off for positive values of high sensitivity troponin varies by sex with lower threshold in women than in men to diagnose ischemia.
   4. Differential considerations: Takatsubo’s Cardiomyopathy, Spontaneous coronary artery dissection.
   5. Vasoreactive disease or syndrome X occurs more commonly in women and involves both large arteries (coronary artery vasospasm) or small arteries (coronary artery embolization or coronary microvascular dysfunction); can also occur with nonobstructive CAD explaining lower rates of obstruction on angiography in women than men (Women’s Ischemia Syndrome Evaluation)
5. Pathophysiology
   1. Higher rates of vasoreactive dysfunction with microvascular disease and endothelial dysfunction in women than men relative to typical obstructive lesions
6. **Management**
   1. PCI as indicated for obstructive CAD, though women were found to have higher risk of adverse events if they undergo early invasive treatment in presence of negative biomarkers
   2. Dosing of anticoagulant in women needs to be adjusted lower based on ideal body weight – otherwise associated with higher risk of bleeding
   3. Treatment of ischemia in presence of nonobstructive CAD is geared towards symptom management and vascular function improvement and identification and treatment based on the underlying cause (vasospasm, coronary artery embolization, coronary artery dissection or microvascular dysfunction).
7. Prognosis
   1. Women have higher morbidity and mortality after an MI than men, more so with STEMI and women < 50 years
   2. Women with non-obstructive CAD have higher adverse event rates than those with normal coronaries unlike men

**References:**

Benn et al. Extreme concentrations of endogenous sex hormones, ischemic heart disease, and death in women. [Arterioscler Thromb Vasc Biol](javascript:AL_get(this,%20'jour',%20'Arterioscler%20Thromb%20Vasc%20Biol.');) 2015;35(2): 471-477.

Daughtery et al. Age-dependent gender differences in hypertension management. J Hypertension. 2011 May;29(5):1005-11.

Hak et al. Systemic lupus erythematosus and the risk of cardiovascular disease: results from the nurses' health study. Arthritis Rheum. 2009 Oct 15;61(10):1396-402.

Huxley et al. Cigarette smoking as a risk factor for coronary heart disease in women compared with men: a systematic review and meta-analysis of prospective cohort studies. Lancet. 2011 Oct 8;378(9799):1297-305

Khan et al. Sex differences in acute coronary syndrome symptom presentation in young patients. JAMA Int Med 2103; 173(20):1863-71.

McSweeney et al. Women's early warning symptoms of acute myocardial infarction. [Circulation.](https://www-ncbi-nlm-nih-gov.revproxy.brown.edu/pubmed/14597589) 2003 Nov 25;108(21):2619-23

Marroquin et al. Metabolic syndrome modifies the cardiovascular risk associated with angiographic coronary artery disease in women: a report from the Women's Ischemia Syndrome Evaluation. Circulation. 2004 Feb 17;109(6):714-21.

Rutledge et al. Depression symptom severity and reported treatment history in the prediction of cardiac risk in women with suspected myocardial ischemia: The NHLBI-sponsored WISE study. Arch Gen Psych. 2006 Aug;63(8):874-80.

Shaw et al. Women and ischemic heart disease: evolving knowledge. J Am Coll Cardiology 2009; 54(17):1561-75.

Soffler et al. It’s not all chest pain: sex and gender in acute care cardiology. Sex and Gender in Acute Care Medicine. Cambridge University Press. 2016

Ting et al. Delay from symptom onset to hospital presentation for patients with non-ST-segment elevation myocardial infarction. [Arch Intern Med.](https://www-ncbi-nlm-nih-gov.revproxy.brown.edu/pubmed/21059977) 2010 Nov 8;170(20):1834-41.

Towfighi et al. Sex-specific trends in midlife coronary heart disease risk and prevalence.

Arch Intern Med. 2009 Oct 26;169(19):1762-6

Vaccarino et al. Sex-based differences in early mortality after myocardial infarction. National Registry of Myocardial Infarction 2 Participants. NEJM 1999 Jul 22;341(4):217-25.

Vaccarino. Ischemic Heart Disease in Women: Many Questions, Few Facts. [Circ Cardiovasc Qual Outcomes. 2010 Mar; 3(2): 111–115.](https://www-ncbi-nlm-nih-gov.revproxy.brown.edu/entrez/eutils/elink.fcgi?dbfrom=pubmed&retmode=ref&cmd=prlinks&id=20160161)
