# Supplementary material for: Integrating Sex and Gender into an Interprofessional Curriculum: Workshop Proceedings from the 2018 Sex and Gender Health Education Summit
Source: J Womens Health (Larchmt). 2019 Dec 10;28(12):1737–42. doi: 10.1089/jwh.2018.7339 (PMC6919237; doi:10.1089/jwh.2018.7339)
Supplement: Supplemental data [file Supp_Appendix2.docx]

**GROUP FACILITATOR GUIDE**

**WORKSHOP A: What Does Sex and Gender Educational Integration Look Like in the**

**Classroom?**

**Goals/Objectives for the activity:**

Using an interdisciplinary and interprofessional cohort, the goals of this workshop are to:

1. Analyze common clinical scenarios that highlight the sex and gender differences in presentation, diagnosis or management of illness.

2. Utilize educational tools that promote active learning to teach sex and gender based medicine

clinical pearls to diverse learner audiences

3. Determine highest yield instructional modalities to instruct trainees in health professions about

sex and gender’s influence on clinical care.

**Overview:** You will be partnered as a discussion facilitator with a group table of approximately 8-12 attendees. The group will be given 2 clinical cases that have been written to be blind to both sex and gender. Each case contains discussion points based on evidence that highlight sex and gender differences in general areas such as risk factors and epidemiology, patient presentation and diagnosis, pathogenesis of disease, and treatment and clinical management. Each table will also have standard definitions of a selection of instructional and assessment methods based on the AAMC curriculum inventory for reference during the session.

**Facilitator Role**: Most attendees likely will be health professions educators, but may have wide variation in their knowledge of sex and gender based medicine. Encourage the group to walk through the clinical case and identify some of the evidence-based discussion points that resonate or surprise them. The group’s task will be to take some of those discussion points and develop a plan to deliver that knowledge—as well as assess whether delivery has been successful—through standardized instructional and assessment methods.

• **The group collectively should attempt to write 2-3 SMART (specific, measurable, attainable, relevant, and time-framed) learning objectives that address the sex and**

**gender points of interest. Then they should develop a framework for how the**

**information would be delivered in instruction**. For example: “Women have more T-wave

inversions and a potentially lower troponin,” when presenting with an NSTEMI. This could be

delivered through a *problem-based learning* activity involving EKG interpretation, which

could be assessed through an EKG OSCE, a standardized simulation patient, or an institutional

EKG exam.

• **Challenge the group to use instructional methods that facilitate active learning.** One aim of this workshop is to push educators to expand their intentional use of less conventional

instructional methods such as journal clubs, peer-teaching, and games. You have a few choices

in terms of how to do this. You can assign specific evaluation and assessment methods for both cases, allow attendees to choose from specific methods for one or both cases, or allow them free reign in terms of choosing methods. It is suggested that you be prepared to suggest some specific methods in case your group becomes sidetracked in choosing methods.

• **Encourage discussion about how sex and gender fits into interprofessional education for the health professions**. Attendees will be present from many health professions, including but not limited to medicine, dentistry, pharmacy, nursing, and public health.

• **Ask the group to share barriers and solutions to integrating sex and gender into their instructional methods within the classroom**. Consider institutional resources, faculty

availability, and how sex and gender may fit into the overarching curriculum.
