# Supplementary material for: Integrating Sex and Gender into an Interprofessional Curriculum: Workshop Proceedings from the 2018 Sex and Gender Health Education Summit
Source: J Womens Health (Larchmt). 2019 Dec 10;28(12):1737–42. doi: 10.1089/jwh.2018.7339 (PMC6919237; doi:10.1089/jwh.2018.7339)
Supplement: Supplemental data [file Supp_Appendix3.docx]

**Developing SMART Objectives**

One way to develop well-written objectives is to use the SMART approach. Developing specific, measurable objectives requires time, orderly thinking, and a clear picture of the results expected from program activities. The more specific your objectives are, the easier it will be to demonstrate success.

SMART stands for Specific Measurable Attainable/Achievable Relevant

**Specific**—What exactly are we going to do for whom? The “specific” part of an objective tells us what will change for whom in concrete terms. It identifies the population or setting, and specific actions that will result. In some cases it is appropriate to indicate how the change will be implemented (e.g., through training). Coordinate, partner, support, facilitate, and enhance are not good verbs to use in objectives because they are vague and difficult to measure. On the other hand, verbs such as provide, train, publish, increase, decrease, schedule, or purchase indicate clearly what will be done.

**Measurable**—Is it quantifiable and can WE measure it? Measurable implies the ability to count or otherwise quantify an activity or its results. It also means that the source of and mechanism for collecting measurement data are identified, and that collection of these data is feasible for your program or partners.

A baseline measurement is required to document change (e.g., to measure percentage increase or decrease). If the baseline is unknown or will be measured as a first activity step, that should be indicated in the objective as “baseline to be determined using XXX database, 20XX.” The data source you are using and the year the baseline was obtained should always be specified in your objective statement. If a specific measurement instrument is used, you might want to incorporate its use into the objective.

Another important consideration is whether change can be measured in a meaningful and interpretable way given the accuracy of the measurement tool and method.

**Attainable/Achievable**—Can we get it done in the proposed time frame with the resources and support we have available?

The objective must be feasible with the available resources, appropriately limited in scope, and within the program’s control and influence.

Sometimes, specifying an expected level of change can be tricky. To help identify a target, talk with an epidemiologist, look at historical trends, read reports or articles published in the scientific or other literature, look at national expectations for change, and look at programs with similar objectives. Consult with partners or stakeholders about their experiences. Often, talking to others who have implemented similar programs or interventions can provide you with information about expected change.

In some situations, it is more important to consider the percentage of change as a number of people when discussing impact. Will the effort required to create the amount of change be a good use of your limited resources?

**Relevant**—Will this objective have an effect on the desired goal or strategy? Relevant relates to the relationship between the objective and the overall goals of the program or purpose of the intervention. Evidence of relevancy can come from a literature review, best practices, or your theory of change.

**Time bound**—When will this objective be accomplished? A specified and reasonable time frame should be incorporated into the objective statement. This should take into consideration the environment in

which the change must be achieved, the scope of the change expected, and how it fits into the overall

work plan. It could be indicated as “By December 2010, the program will” or “Within 6 months of receiving the grant,...”

**Using SMART Objectives**

Writing SMART objectives also helps you to think about and identify elements of the evaluation plan and measurement, namely indicators and performance measures.

An indicator is what you will measure to obtain observable evidence of accomplishments, changes made, or progress achieved. Indicators describe the type of data you will need to answer your evaluation questions. A SMART objective often tells you what you will measure.

A performance measure is the amount of change or progress achieved toward a specific goal or objective. SMART objectives can serve as your performance measures because they provide the specific information needed to identify expected results.

**Getting Started**

To develop SMART objectives, use the template below and fill in the blanks:

By / / ,

[WHEN—Time

bound] [WHO/WHAT—Specific]

from to

[MEASURE

(number, rate, percentage of change and baseline)—Measurable]

Adapted from:

<http://www.kean.edu/sites/default/files/u7/SMART%20Objectives.pdf>

<http://www.cdc.gov/dhdsp/state_program/evaluation_guides/pdfs/smart_objectives.pdf>
